# Deep-learning-based image quality enhancement of compressed sensing magnetic resonance imaging of vessel wall: comparison of self-supervised and unsupervised approaches

**DOI:** 10.1038/s41598-020-69932-w

**Published:** 2020-08-18

**Authors:** Da-in Eun, Ryoungwoo Jang, Woo Seok Ha, Hyunna Lee, Seung Chai Jung, Namkug Kim

**Affiliations:** 1 Department of Convergence Medicine, University of Ulsan College of Medicine, Asan Medical Center, 88 Olympic-ro 43-gil, Songpa-gu, Seoul, South Korea; 2 School of Medicine, Kyunghee University, 26-6, Kyungheedae-ro, Dongdaemun-gu, Seoul, South Korea; 3 Department of Neurology, Yonsei University College of Medicine, 50, Yonsei-ro, Seodaemun-gu, Seoul, South Korea; 4 Department of Radiology and Research Institute of Radiology, University of Ulsan College of Medicine, Asan Medical Center, 88 Olympic-ro 43-gil, Songpa-gu, Seoul, South Korea

**Keywords:** Image processing, Translational research, Magnetic resonance imaging

## Abstract

While high-resolution proton density-weighted magnetic resonance imaging (MRI) of intracranial vessel walls is significant for a precise diagnosis of intracranial artery disease, its long acquisition time is a clinical burden. Compressed sensing MRI is a prospective technology with acceleration factors that could potentially reduce the scan time. However, high acceleration factors result in degraded image quality. Although recent advances in deep-learning-based image restoration algorithms can alleviate this problem, clinical image pairs used in deep learning training typically do not align pixel-wise. Therefore, in this study, two different deep-learning-based denoising algorithms—self-supervised learning and unsupervised learning—are proposed; these algorithms are applicable to clinical datasets that are not aligned pixel-wise. The two approaches are compared quantitatively and qualitatively. Both methods produced promising results in terms of image denoising and visual grading. While the image noise and signal-to-noise ratio of self-supervised learning were superior to those of unsupervised learning, unsupervised learning was preferable over self-supervised learning in terms of radiomic feature reproducibility.

## Introduction

Compressed sensing in magnetic resonance imaging (MRI) is a commonly used signal processing technology for efficiently acquiring and reconstructing an MR signal. It is based on the principle that the sparsity of a signal can be exploited to recover it from significantly fewer samples than are required by the Shannon–Nyquist sampling theorem^1,2^. Along with sensitivity encoding using a receiver array (SENSE)^3^, compressed sensing technologies have also been introduced to reduce the MR scan time further^4^. Compressed SENSE (CS) that concurrently uses compressed sensing and sensitivity encoding has become the next generation MR technology, as it can significantly reduce the scan time of an MRI.

Among the MRI sequences commonly used for investigations on vessel wall, high-resolution proton density-weighted (HRPD) MRI of the intracranial vessel wall is significant, as it can provide excellent anatomic information on even tiny vascular structures based on high signal-to-noise ratios, thus providing precise diagnoses of intracranial artery disease and guidance in terms of the appropriate treatment decision^5^. Though there are other choices such as the T1 weighted image, PD is preferable as it can achieve a high signal-to-noise ratio^6^.

However, acquiring HRPD MR images takes a long time, particularly when using a spatial resolution of ≤ 0.4 mm^3^, which could cause patient discomfort and motion artefacts. To address this problem, CS can be applied to reduce the scan time while preserving image quality to some extent^7,8^. However, loss of image quality, as compared with a fully sampled MRI, is unavoidable. A previous study^9^ reported that CS HRPD MR images with high acceleration factors yields unacceptable imaging for radiological reading. As low-quality vessel wall imaging hampers accurate diagnosis^9–11^, improving the image quality of CS HRPD MR images would be beneficial for accurate radiological reading and diagnosis.

Furthermore, the significance of improving image quality is increasing for data-driven computer-based medical imaging research, as the information extracted from the images is impacted by noise^12–14^. Particularly in radiomics, which extracts quantitative features from medical images that may exhibit valuable medical information, reproducibility and generalisability is one of the biggest challenges^13,14^. Image quality difference among MRI images that are acquired using different imaging protocols is one of the factors the cause generalisability issues^15,16^. Therefore, improving image quality while preserving imaging features is essential in terms of image standardisation and generalisation.

Recently, image quality enhancement using a convolutional neural network (CNN) that aims to restore the corrupted, noisy images to clean images has achieved significant success in computer vision. In most cases, the image restoration networks were trained with corrupted–uncorrupted image pairs that are aligned pixel-wise and directly minimise the mean squared error (MSE) or absolute error between the input and output images of the network. However, this approach is not applicable for medical images in a routine clinical process, as it is difficult to collect real pixel-wise clean and unclean pairs of MR images owing to motion artefacts unless fully sampled k-space data is stored.

Similar issues also exist in low-dose computed tomography (CT) enhancement tasks. For instance, cardiac CT images at low- and high-dose phases have pixel misalignment problems due to cardiac motion. Therefore, respiratory motion artifacts may cause pixel-alignment problems between low-dose and high-dose CT images^17,18^.

To address this lack of training pairs in medical imaging, two deep-learning (DL)-based approaches offer the following possibilities: (1) pixel-wise training pairs can be obtained by artificially generating corrupted medical images^19,20^ or (2) unsupervised image translation networks can be applied based on generative adversarial networks (GAN)^17,21^.

In this study, we compared the network output images of two different DL-based approaches in terms of (1) quantitative and qualitative image quality enhancement and (2) imaging feature reproducibility for radiomics. In the first approach, we synthesised pixel-wise alignment training pairs by adding random Gaussian noise to clean MR images^22^. Thereafter, the network was trained for denoising the noisy high-acceleration CS HRPD MR images in the inference phase, as a self-supervised learning method. In the second approach, we utilised cycle-consistent adversarial networks^17^ to transfer noisy MR images to clean MR images, as an unsupervised learning method.

In summary, the main contributions of this paper are as follows:

1. We successfully reduced the noise in CS HRPD MR images using two different DL-based methods. Thereafter, denoising methods can further reduce the HRPD MR scan time with a lower compromise on image quality. Furthermore, the proposed methods are expected to be applied to other medical image restoration tasks without the pixel-wise alignment of training pairs.
2. This is the first study that directly compares two different DL-based image restoration methods. Therefore, we expect that this study will facilitate setting guidelines for choosing the appropriate image restoration network depending on the purpose.

## Related works

Accelerating MRI acquisition is a topic that has gained significant attention in literature. Currently, parallel imaging hardware and compressed sensing MRI are frequently used in real clinical settings for fast MRI acquisition process. However, these reconstruction algorithms require multiple prior information. For instance, parallel MRI using coil sensitivity information and CS MRI reconstruction utilizes image sparsity as a prior for fast MRI reconstructions. With the recent advances in DL, several efforts have been made on fast MRI acquisition using DL-based methods. Compared with traditional methods, DL-based methods do not require enormous amounts of prior information. The DL-based method for fast MRI acquisition can be classified into four categories^23^: (1) Denoising acquired MRI images in the image domain^24,25^, (2) Updating both k-space domain and image domain using cascaded DL^26–29^, (3) Direct conversion of k-space data to image domain data through DL^30^, and (4) Interpolation of missing k-space data through DL and obtaining images through inverse Fourier transform^23^. Our study falls under category (1), which attempts to denoise the images in the post-processing steps. The direct use of the image domain, compared with k-space data, is advantageous, as it does not require additional information regarding k-space and coils. Furthermore, it has the potential to be used in other medical imaging devices other than MRI^25^.

## Materials and methods

### Image acquisition

The institutional review board for human investigations at Asan Medical Center (AMC) approved this study. Informed consent was obtained from all subjects. The research was performed in accordance with relevant guidelines and regulations. The study population and image acquisitions were as previously described^9^.

#### Study population

Fourteen healthy volunteers, including 7 men (mean age, 58.6 years; age range, 40–67 years) and 7 women (mean age, 55.3 years; age range, 33–65 years) with a general age of 56.6 years (age range, 33–67 years) were included in this study. All subjects underwent HRPD MRI for evaluation of intracranial arteries.

#### Image acquisition

**Figure 1:**
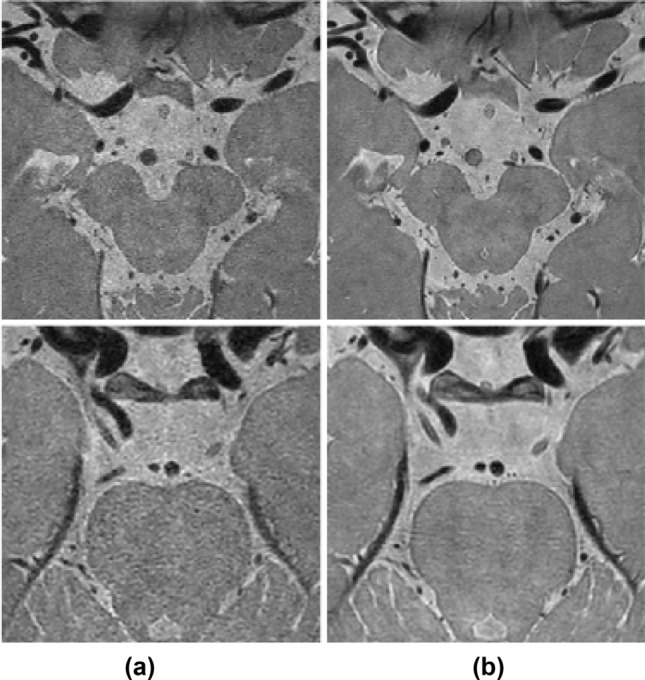
Example of dataset. Our dataset consists of (**a**) compressed SENSE (CS) AFt 5.8 and (**b**) original images (SENSE AFt 2.0).

A 3-T MRI system (Ingenia CX, Philips Medical System, Best, the Netherlands) with a 32-channel sensitivity-encoding head coil was used for all MR examinations. For delineation of the intracranial arteries, three-dimensional (3D) proton density-weighted imaging (PD) was carried out. CS HRPD MR images with a high acceleration factor (AF_t_ 5.8) and original imaging sequence (SENSE PD with AF_t_ 2.0) were acquired. The scan parameters are presented in Table 1. An example of the acquired dataset is displayed in Fig. 1.

**Table 1:** Scan parameters of acquired proton density-weighted MR scans.

	CS AF_t_ 5.8	Original
MRI acceleration technique	Compressed SENSE	SENSE
Scan duration	4 min 22 s	12 min 36 s
Extra-reduction factor	2.9	–
Acceleration factor (AF_t_)	5.8	2.0
Echo time (ms)	35	35
Repetition time (ms)	2000	2000
Flip angle (°)	90	90
Matrix	300 × 300 × 75	300 × 300 × 75
Field of view (FOV) (mm)	120 × 120 × 30	120 × 120 × 30
Voxel size (mm)	0.4 × 0.4 × 0.4	0.4 × 0.4 × 0.4
Sequence	3D TSE	3D TSE
Sampling Pattern	Radial-Cartesian	Radial-Cartesian
Reconstruction algorithm	Compressed sensing algorithm work in progress (Philips)	SENSE algorithm work in progress (Philips)

### General frameworks

Image denoising is a process that converts corrupted (noisy) input images into uncorrupted (clean) output images. This study aims to determine the mapping function for the transformation of a corrupted image into a clean image. Recently, deep convolutional neural networks (CNN) have performed well in determining the mapping function for image restoration^31–33^. Training the regression model of CNN is described as

$$ argmin_{\theta } \mathop \sum \limits_{i}^{{}} L\left( {f_{\theta } \left( {x_{i } } \right),y_{i} } \right) $$

where $$L $$ is loss function, $$x_{i}$$ is the corrupted input, $$y_{i }$$ is the uncorrupted target, and $$f_{\theta }$$ is the mapping function with the parameter $$\theta$$.

**Figure 2:**
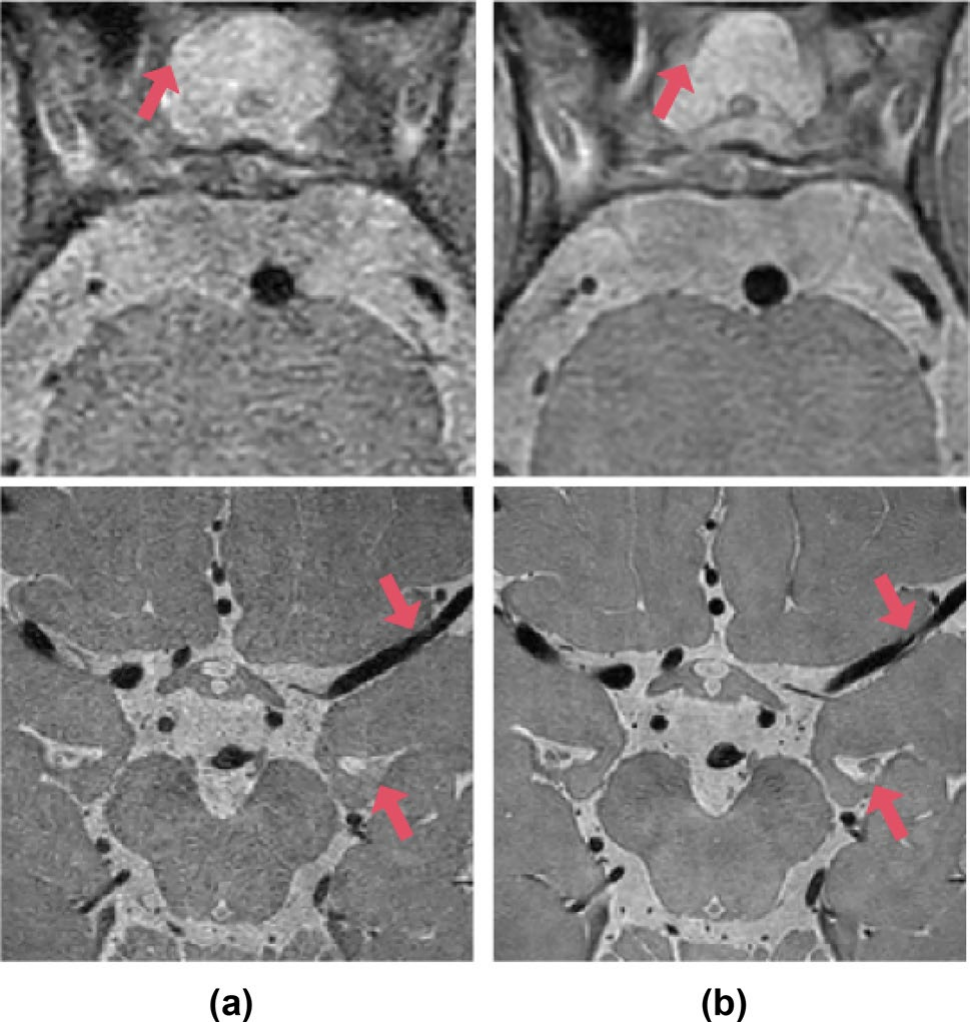
Local structure comparison between (**a**) CS AF_t_ 5.8 and (**b**) original images (SENSE AF_t_ 2.0). Details of local structure, such as cerebrospinal fluid (CSF) and blood vessels, vary between acquired magnetic resonance scans (red arrows).

In our dataset of noisy CS input images and clean original images, $x_{i}$ represents the noisy CS MR images and $y_{i }$ represents the clean original MR images. However, as there was a time interval during the acquisition of these images, the corresponding CS and original images do not have exact pixel-to-pixel alignment. Therefore, the loss function $L$ (typically L1 loss or L2 loss) could not represent the distance between the CS and original images in the manifold domain, as the pixel distance was inconsistent with the perceptual distance^34^. As presented in Fig. 2, the local structure of images acquired at the same position varied significantly between the acquired scans. As the local structure variations and other factors that resulted from the imaging acquisition process contributed significantly to the Euclidean pixel distance, the mean squared error and absolute error do not represent the differences in perceptual noise level. Therefore, we used two different DL-based image denoising methods to address this problem, as described in "Self-supervised learning" and "Unsupervised learning".

#### Self-supervised learning

In self-supervised learning, we artificially generated corrupted input images $\widehat{{x_{i} }}$, instead of directly using CS AF_t_ 5.8 MR images as corrupted inputs, to preserve the pixel-wise alignment between the artificially generated input and target images. The corrupted MRI input images can be obtained in two different ways: (1) undersampling of k-space data or (2) corrupting the sampled data by adding noise. In both approaches, it is difficult to completely understand the noise distribution of the reconstructed MR images because of the black box nature of the acquisition process and its influence on the noise distribution^35,36^. In this study, we used the second approach for generating artificial inputs. The comparison between the two different noise modelling approaches is discussed in “Comparison of artificial noise modelling methods” section.

In general, it is well known that noise in the reconstructed MR image from a single coil follows Rician noise distribution^37^. Therefore, many MRI denoising studies are based on Rician noise distribution^38–40^. For instance^38^, successfully developed MRI Rician noise removal algorithm using spatially adaptive constrained dictionary learning. However, the nature of MRI noise became more complex as modern MR techniques, in particular, diverse parallel imaging techniques have been developed. The noise statistics of multi-coil parallel imaging MRI is still not fully understood and experimentally explored^36^. Therefore, we simply approximated the noise distribution as random Gaussian noise, as the noise distribution of multi-coil MR images can be approximated as random Gaussian noise^22,41–44^. Since we have obtained real corrupted CS AF_t_ input images, we analysed noise distribution of CS 5.8 AF_t_ images. The noise was measured in the air-filled anatomical region (e.g., a nasal cavity of size 10 × 10 matrices). A normality test was conducted based on D’Agostino and Pearson’s test^45,46^, using the SciPy normal test package. According to the results of the normality test, the noise distribution follows a normal distribution with *P* value > 0.01. Therefore, four our experiment, we approximated the noise model as a Gaussian noise model as follows :

1 $$ \widehat{{x_{j} }} = y_{i} + g\left( {\mu ,\sigma } \right) $$

where $$\widehat{{x_{i} }}$$ is the artificially generated corrupted input, $$y_{i} $$ is the clean target, and $$g\left( {\mu ,\sigma } \right)$$ is Gaussian noise with mean $$\mu$$ and standard deviation $$\sigma$$.

**Figure 3:**
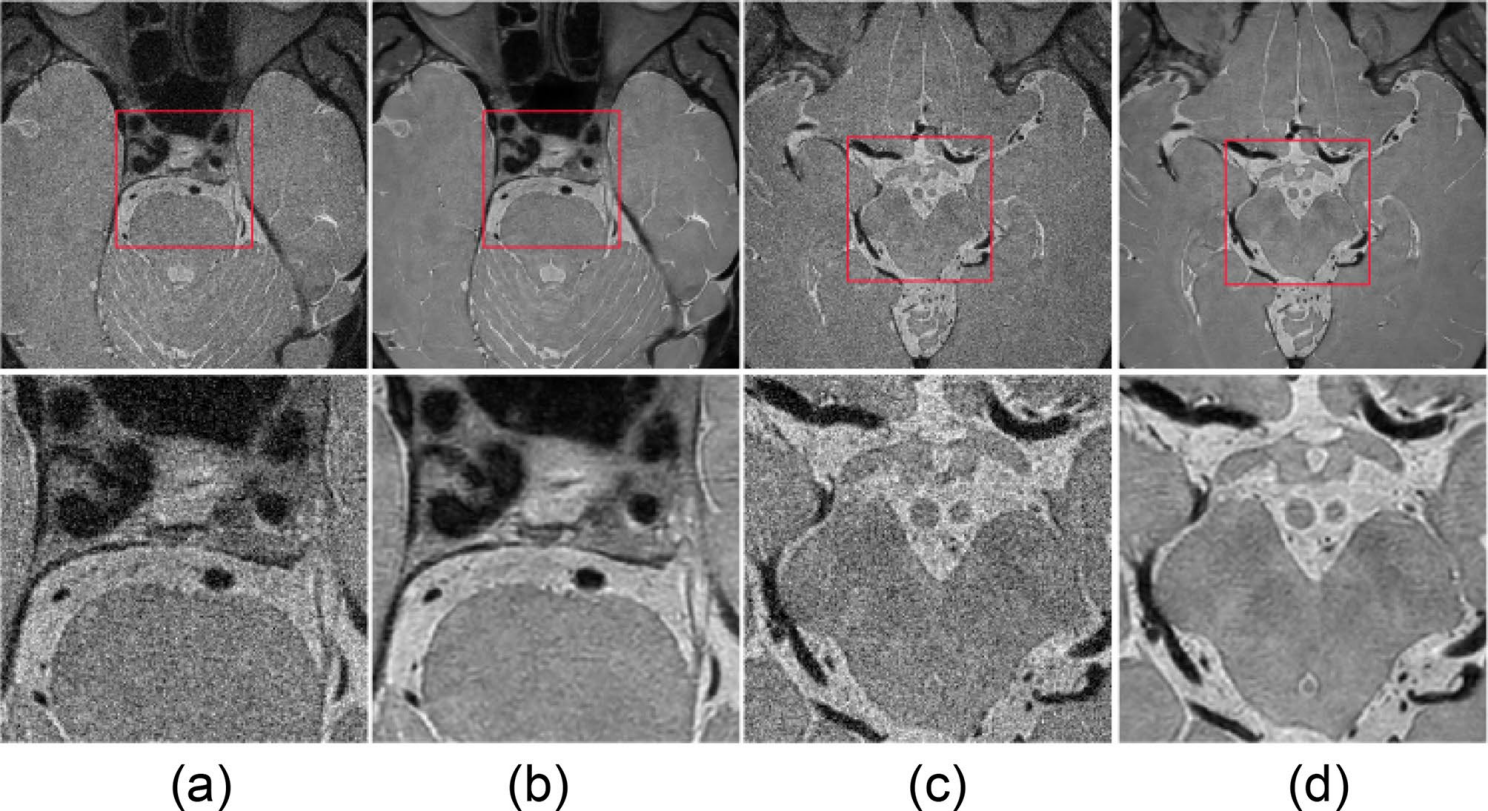
Target original images (**b**, **d**) and artificial input images (**a**, **c**). Region of interest that contains basilar artery (left two columns) and middle cerebral artery (right two columns) inside red box are enlarged in lower column.

The parameter $\mu$ was set to zero, with standard deviation ranges from 10 to 50% of the arithmetic mean. We selected the standard deviation range that gives the best network performance, i.e., the one which is 25–35% of the arithmetic mean. Figure 3 displays the target (original) and artificially generated input images. With the artificial corrupted images, the regression problem in the training phase can be written as follows:

2 $$ argmin_{\theta } \mathop \sum \limits_{i}^{{}} L\left( {f_{\vartheta } \left( {\widehat{{x_{i} }}} \right),y_{i} } \right) $$

where $$\widehat{{x_{i} }}$$ is the artificially generated corrupted input and $$y_{i} $$ is the clean target.

**Figure 4:**
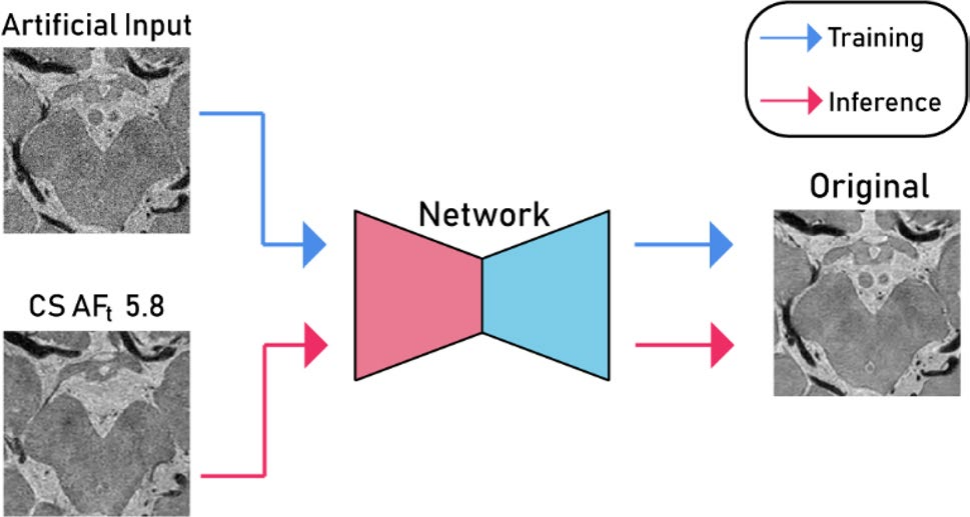
Basic implementation of self-supervised learning (encoder network, red; decoder network, cyan).

After training, inference is a simple feed-forward problem. In the inference phase, we replaced the artificial input with CS AF_t_ 5.8 input images to dispose of noise in the real CS images (Fig. 4).

**Figure 5:**
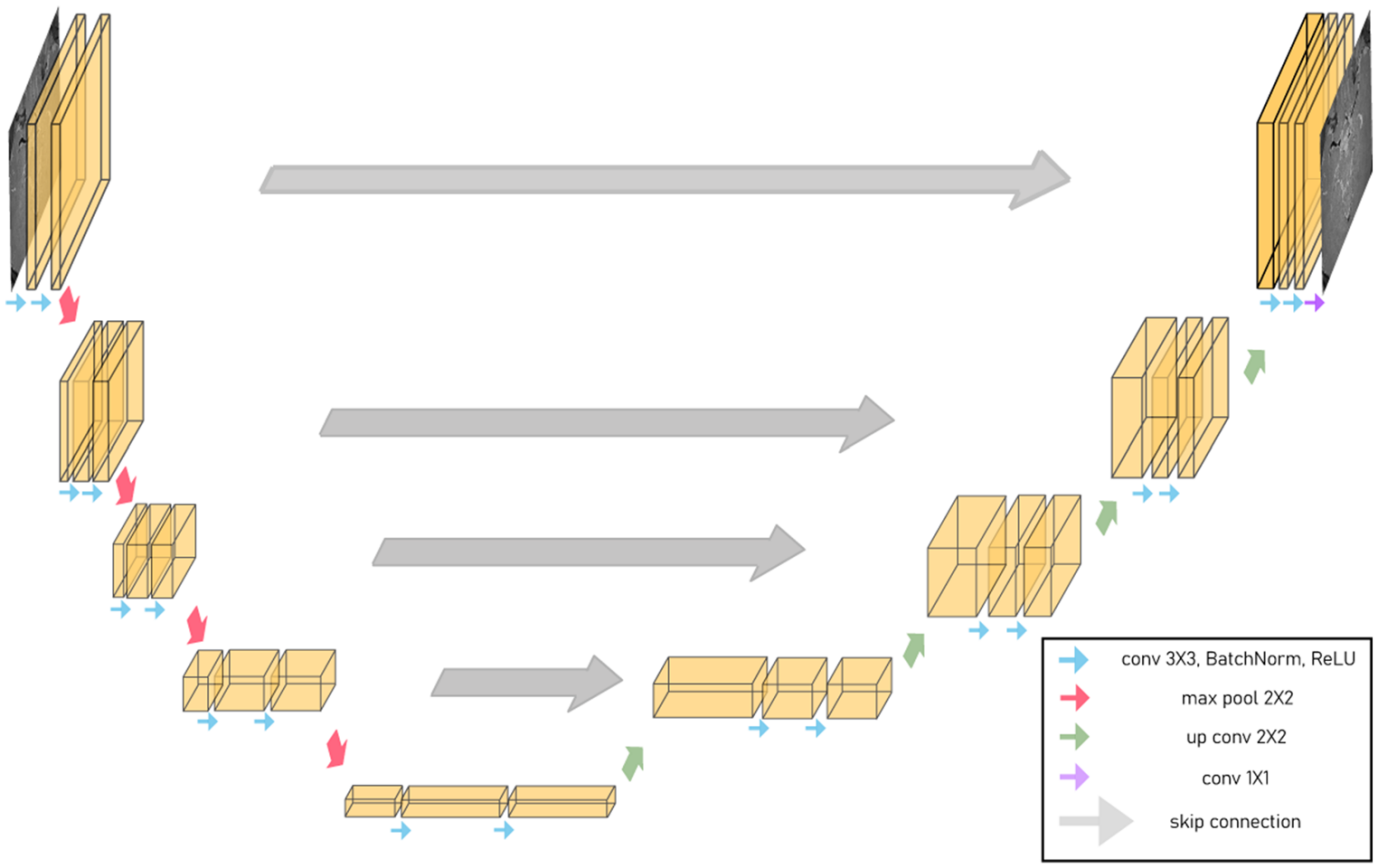
Denoising network architecture of U-Net used for self-supervised learning.

Recent deep CNN has employed encoder–decoder architecture for identifying the denoising algorithm. U-Net is a convolutional auto-encoder with skip connections^47^, which can capture high-level image details and reproduce it from corrupted images. Therefore, we utilised the basic structure of U-Net^47^ as a denoising network. A 2 × 2 max pooling layer was used for the down-sampling and a 2 × 2 transpose convolution layer was used for the up-sampling. In addition, w e added a batch normalisation layer before the activation layer and both the input and output channels were a single channel (Fig. 5).

#### Unsupervised learning

**Figure 6:**
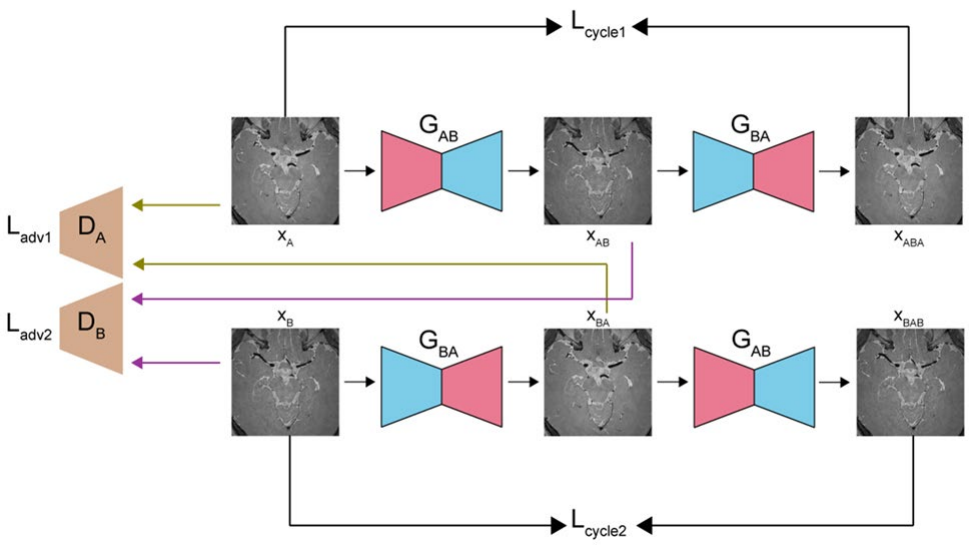
Basic implementation of CycleGAN for unsupervised learning.

In unsupervised learning, we implemented the cycle generative adversarial network (CycleGAN)^17^. The objective of CycleGAN is to map domain A to domain B and vice versa. With domain A as input images (CS AF_t_ 5.8) and domain B as target images (original), the generators *G*_AB,_ which maps A to B, and *G*_BA_, which maps B to A, are used with the discriminators *D*_A_, which discriminates real images from domain A and generated images, and *D*_B_, which discriminates real images from domain B and generated images (Fig. 6). The generators and discriminators were trained simultaneously. While pixel alignment was required in self-supervised learning, as the network was directly trained between input and target images using L1 loss, the loss functions of CycleGAN do not require pixel-to-pixel alignments. Therefore, pixel-misalignment problems can be eased with the unsupervised learning approach using CycleGAN. The optimisation problem is as follows^17,48^:

$$ \mathop {\min }\limits_{{G_{AB} , G_{BA} }} \mathop {\max }\limits_{{D_{A} ,D_{B} }} L\left( {G_{AB} , G_{BA} ,D_{A} , D_{B} } \right) $$

where

3 $$ L\left( {G_{AB} ,G_{BA} ,D_{A} ,D_{B} } \right) = L_{GAN} \left( {G_{AB} ,D_{B} ,A,B} \right) + L_{GAN} \left( {G_{BA} ,D_{A} ,B,A} \right) + \lambda L_{cyclic} \left( {G_{AB} ,G_{BA} } \right) $$

In further detail, the min–max problem of GAN loss with MSE can be represented as follows^49^:

$$ \begin{aligned} & \mathop {min}\limits_{{G_{AB} }} \mathop {max}\limits_{{D_{B} }} L_{GAN} \left( {G_{AB} , D_{B} ,A , B} \right) \\ & = \mathop {min}\limits_{{G_{AB} }} \left[ {\left( {1 - D_{B} \left( {G_{AB} \left( {x_{A} } \right)} \right) ^{2} } \right)} \right],\;\mathop {min}\limits_{{D_{B} }} {\mathbb{E}}_{{x_{B} \sim P_{B} }} \left[ {\left( {D_{B} \left( {x_{B} } \right) - 1} \right)^{2} } \right] \\ & \quad + {\mathbb{E}}_{{x_{A} \sim P_{A} }} \left[ {D_{B} \left( {G_{AB} \left( {x_{A} } \right)} \right) ^{2} } \right] \\ \end{aligned} $$

4 $$ \begin{aligned} & \mathop {min}\limits_{{G_{BA} }} \mathop {max}\limits_{{D_{A} }} L_{GAN} \left( {G_{BA} ,D_{A} ,B,A} \right) \\ & = \mathop {min}\limits_{{G_{BA} }} \left[ {\left( {1 - D_{A} \left( {G_{BA} \left( {x_{B} } \right)} \right) ^{2} } \right)} \right],\;\mathop {min}\limits_{{D_{A} }} {\mathbb{E}}_{{x_{A} \sim P_{A} }} \left[ {\left( {D_{A} \left( {x_{A} } \right) - 1} \right)^{2} } \right] + {\mathbb{E}}_{{x_{B} \sim P_{B} }} \left[ {\left( {1 - \left( {G_{BA} \left( {x_{B} } \right)} \right)} \right) ^{2} } \right] \\ \end{aligned} $$

Through the min–max optimisation, the generators attempt to learn the mapping functions of domain A to B (or B to A) that are sufficient enough to fool the discriminators, which attempt to classify the generated image from the real image to the best extent possible. The cyclic loss is defined as follows:

5 $$ L_{cyclic} \left( {G_{AB} , G_{BA} } \right) = {\mathbb{E}}_{{x_{A} \sim P_{A} }} \left[ {G_{BA} \left( {G_{AB} \left( {x_{A} } \right)} \right) - x_{A} } \right] + {\mathbb{E}}_{{x_{B} \sim P_{B} }} \left[ {G_{AB} \left( {G_{BA} \left( {x_{B} } \right)} \right) - x_{B} } \right] $$

**Figure 7:**
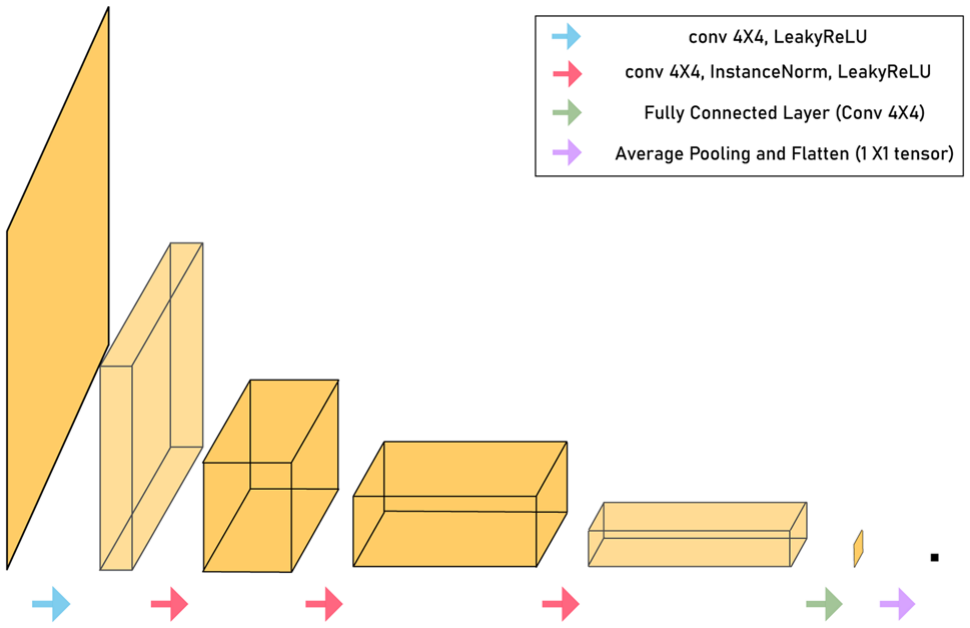
Discriminator architecture of unsupervised learning.

For the generator network, we utilised the same U-Net architecture (Fig. 5) as in self-supervised learning. Figure 7 depicts the architecture of the discriminator, which consists of five convolutional layers and a final fully connected layer. Each convolution layer, except the first convolution layer, is followed by an instance normalisation layer and a leaky ReLU layer; the instance normalisation layer is not included in the first layer. After the fully connected layer, the tensor was average-pooled and flattened into a 1 × 1 tensor.

### Training description

In self-supervised learning, the network was trained by minimising a loss function, which is the MSE between the input and output images. For training, we use input patches of size 256 × 256 from a noisy image with the corresponding clean patches. The number of epochs was 200 and the learning rate was set to 0.002. In the training phase, in which the denoising network is trained for artificial noise, 1,500 original MR images and artificial noise MR images were used for training and 300 images were used for validation. In the inference phase, which tests the performance of the denoising network, 300 CS AF_t_ 5.8 MR images were used.

In unsupervised learning, MSE loss was used for GAN loss and L1 loss was used for cyclic loss. For training, we used input images of size 640 × 640. The number of epochs was 200 and the learning rate was set to 0.002. The same CS AF_t_ 5.8 MR images and original images as described in the abovementioned self-supervised learning were used for training, validation, and testing.

### Evaluation metric

The performance of the image denoising networks were evaluated both quantitatively and qualitatively. For the quantitative analysis, the image quality assessment could be divided into two major categories: (1) reference-based evaluation and (2) no-reference evaluation. In our study, reference-based evaluation metrics, such as MSE, peak signal-to-noise ratio, and structural similarity index, could not be applied, as our MR images did not have pixel-wise alignment reference images. Therefore, we evaluated the image quality of the network output images through no-reference evaluation metrics including signal-to-noise ratio (SNR), image noise, and Blind/Referenceless Image Spatial Quality Evaluator (BRISQUE) score. For the qualitative analysis, a neuroradiologist with more than 10 years of experience visually scored the network output MR images without the noise.

For the assessment of the statistical significance of the evaluation metrics, the Kolmogorov–Smirnov test was used to determine the normality of the distribution of the variables. Metrics for the comparison between input and output images, target and output images, and self-supervised and unsupervised output images were compared using paired *t* tests. Values of *P* < 0.05 were considered statistically significant. Statistical analyses were performed using the python SciPy stats package.

#### Image noise and signal-to-noise ratio

We measured image noise and SNR at the blood vessel wall of the middle cerebral, basilar, and internal carotid arteries. The intensity peaks at the blood vessel wall and decreases rapidly in the vessel lumen. Therefore, the signal was measured as the peak intensity in the blood vessel within a small region-of-interest (ROI) at the blood vessel wall, and the noise was obtained as the standard deviation of the vessel lumen (Eq. (6)):

6 $$ {\text{SNR}}_{{{\text{vessel}}\;{\text{wall}}}} = \frac{{{\text{Peak}}\;{\text{Signal}}_{{{\text{wall}}}} }}{{\upsigma _{{{\text{lumen}}}} }} $$

While the intensity of the blood lumen was not uniform within the vessel lumen, we could compare the noise in the vessel lumen because we took the original image as reference. Furthermore, we measured the image noise and SNR at the cerebrospinal fluid (CSF) in the suprasellar cistern, interpeduncular cistern, and brain parenchyma (Eq. (7)).

7 $$ SNR_{CSF} = \frac{{Signal_{CSF} }}{{\sigma_{CSF} }}, \;SNR_{parenchyma} = \frac{{Signal_{parenchyma} }}{{\sigma_{parenchyma} }} $$

Owing to inhomogeneous noise distribution caused by parallel imaging, noise cannot be simply measured in the air^50,51^. Instead, the signal and noise were measured as the mean and standard deviation of ROIs. The same ROIs of the vessel wall, CSF, and parenchyma were drawn for the CS AF_t_ 5.8, original, and output images of the network.

#### BRISQUE

BRISQUE is a no-reference image quality assessment model that uses natural scene statistics in the spatial domain^52^. This model is composed of three steps: (1) extraction of natural scene statistics, (2) calculation of feature vectors, and (3) prediction of image quality score. We utilised a pretrained prediction model provided by Mittal et al.^52^ for predicting the image quality score. The minimum and maximum image scores are 0 and 100, respectively, with a lower image score indicating better image quality. The potential of BRISQUE as an indicator of medical image quality has been reported previously^53^ and it has been used to predict CS MRI reconstruction parameters^54^.

#### Radiomic feature extraction

For radiomic feature extraction, a large ROI of size 1,600 pixels in brain parenchyma was selected rather than vessel wall to avoid structural variation between the CS AF_t_ 5.8 and original images. As the vessel wall is a small region surrounded by brain tissue, it could lead to variabilities in the radiomic features. As the purpose of the radiomics analysis is to investigate the effect of image quality enhancement on feature variations, the same ROI was drawn for the CS AF_t_ 5.8, original, and output images of the network.

Thereafter, Lin’s concordance correlation coefficients (CCCs)^55^ were calculated between the input CS AF_t_ 5.8 and original images, self-supervised output and original images, and supervised output and original images. A higher CCC score means higher reproducibility of radiologic features.

A total of 465 radiomics features were studied. The features include 18 first-order features, 75 texture features, and 380 wavelet features. The first-order features with intensity histograms include intensity range, energy, skewness, kurtosis, maximum, minimum, mean, uniformity, and variance. For texture analysis, the grey level co-occurrence matrix (GLCM), grey level run length matrix (GLRM), grey level size zone matrix (GLZM), neighbouring grey tone difference matrix (NGTDM), and grey level dependence matrix were calculated. For wavelet features, two-dimensional discrete wavelet transforms were performed in four directions: HH, HL, LH, LL, where ‘L’ indicates a low pass filter and ‘H’ refers to a high pass filter. We utilised open source software for radiomic feature analysis, Pyradiomics^56^.

#### Visual scoring

The HRPD MR images were assessed by a neuroradiologist with 10 years of experience, who was blinded to the sequence information. The images were rated for image quality and vessel delineation using a four-point visual scoring system (Table 2).

**Table 2:** Visual scoring system used to evaluate image quality and vessel wall delineation.

Score	Image quality and artifacts	Vessel delineation of outer contour and branching arteries
3	Excellent image quality without artifacts	Vessel is delineated with excellent signal and sharp contrast to the lumen and CSF
2	Good image quality with slight artifact	Vessel is delineated with adequate signal and contrast to lumen and CSF
1	Moderate image quality with moderate artifact	More than 50% of vessel is visible
0	Poor image quality with large artifact	Less than 50% of vessel is visible

## Results

### Comparison of artificial noise modelling methods

The generation of corrupted input images in the k-space domain was common in studies related to fast MRI reconstruction^24–27^. Therefore, we followed the image quality enhancement methods, used on corrupted input images in the k-space domain, suggested by Wang et al.^26^. The method comprises the following steps:

1. The corrupted input images were obtained by undersampling the k-space domain using Poisson disk sampling mask, and then the images were reconstructed with zero-filling.
2. The super-resolution CNN network^57^ that maps the zero-filled MR images to the fully sampled clean images was trained.
3. The network output images were reconstructed through constrained CS-MR reconstruction optimization.

**Figure 8:**
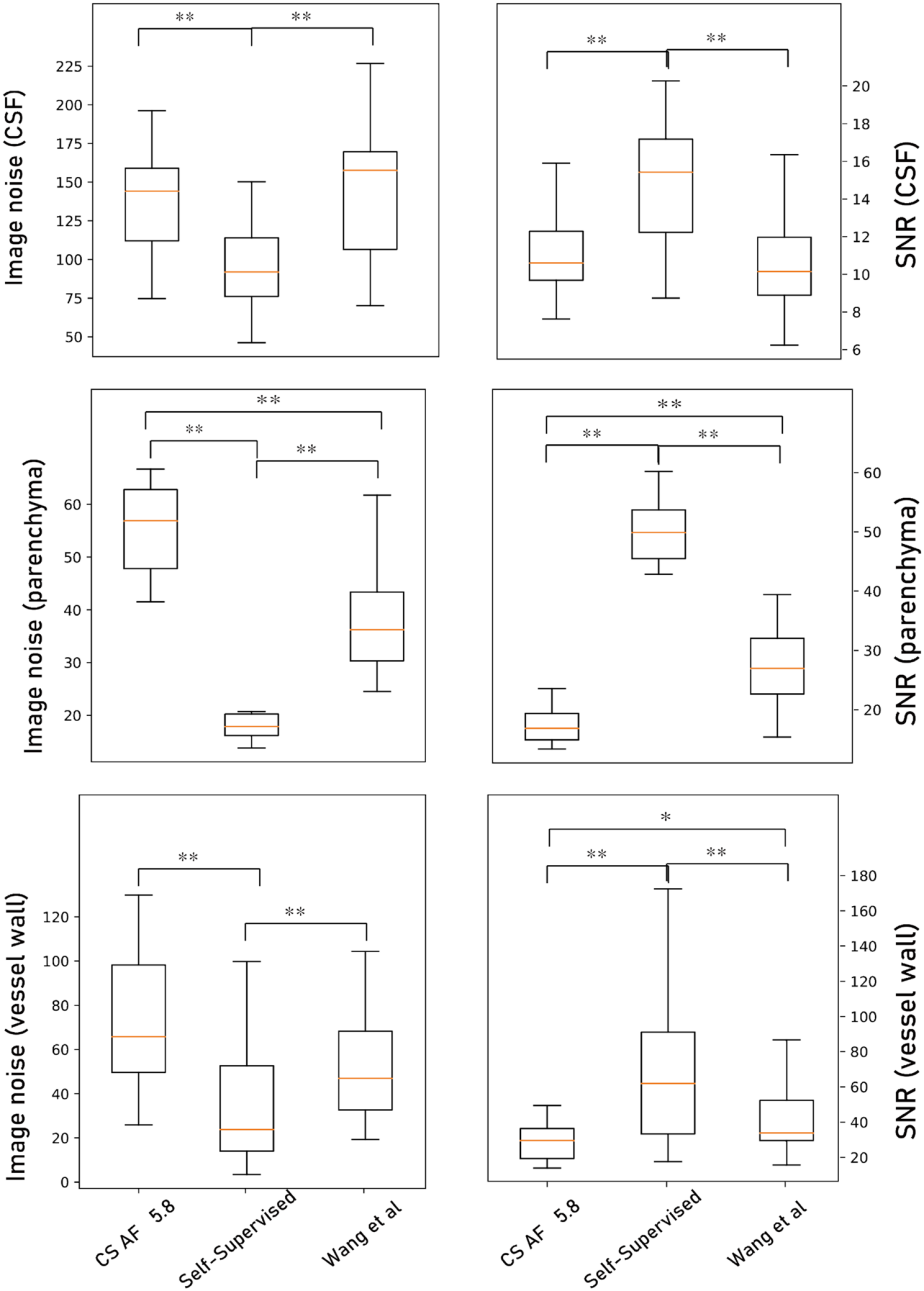
Image noise and signal-to-noise ratio (SNR) results of input CS AF_t_ 5.8 images, target original images, self-supervised learning output images, output images by Wang et al. at the blood vessel wall, CSF, and parenchyma. Paired *t* test results with **P < 0.01 and *P < 0.05.

We followed the abovementioned steps, and then the method was applied to real noisy CS images. For the CS-MRI reconstruction method, we utilized a method suggested by Sparse MRI^58^, SigPy package. Compared with our self-supervised learning method, the noise and SNR measured in vessel wall, CSF, and parenchyma were significantly better in our self-supervised learning method (*P* < 0.01). The results of the comparison are shown in Fig. 8. While different noise modelling methods improved image quality, additive Gaussian noise was more effective in our dataset.

### Comparison of self-supervised learning and unsupervised learning approaches

**Figure 9:**
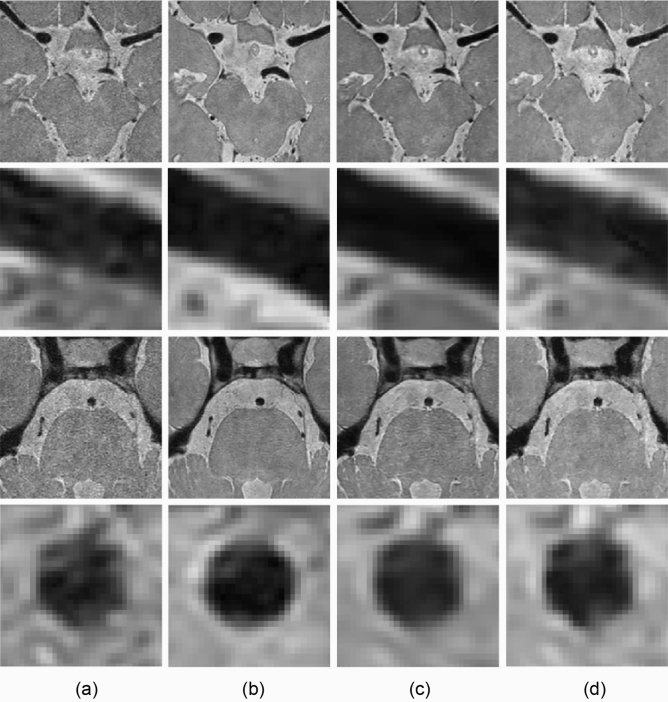
Image denoising results of (**a**) input, (**b**) target (original), and output images of (**c**) self-supervised and (**d**) unsupervised learning approaches. Middle cerebral and basilar arteries are enlarged in the second and fourth rows at a similar position.

The image noise, SNR of the vessel wall and CSF, BRISQUE scores, and radiomic feature reproducibility were measured in the input (CS AF_t_ 5.8), target (original), and output images produced by the self-supervised and unsupervised learning approaches. The results of the denoising are displayed in Fig. 9.

#### Image noise and SNR measurement results

**Figure 10:**
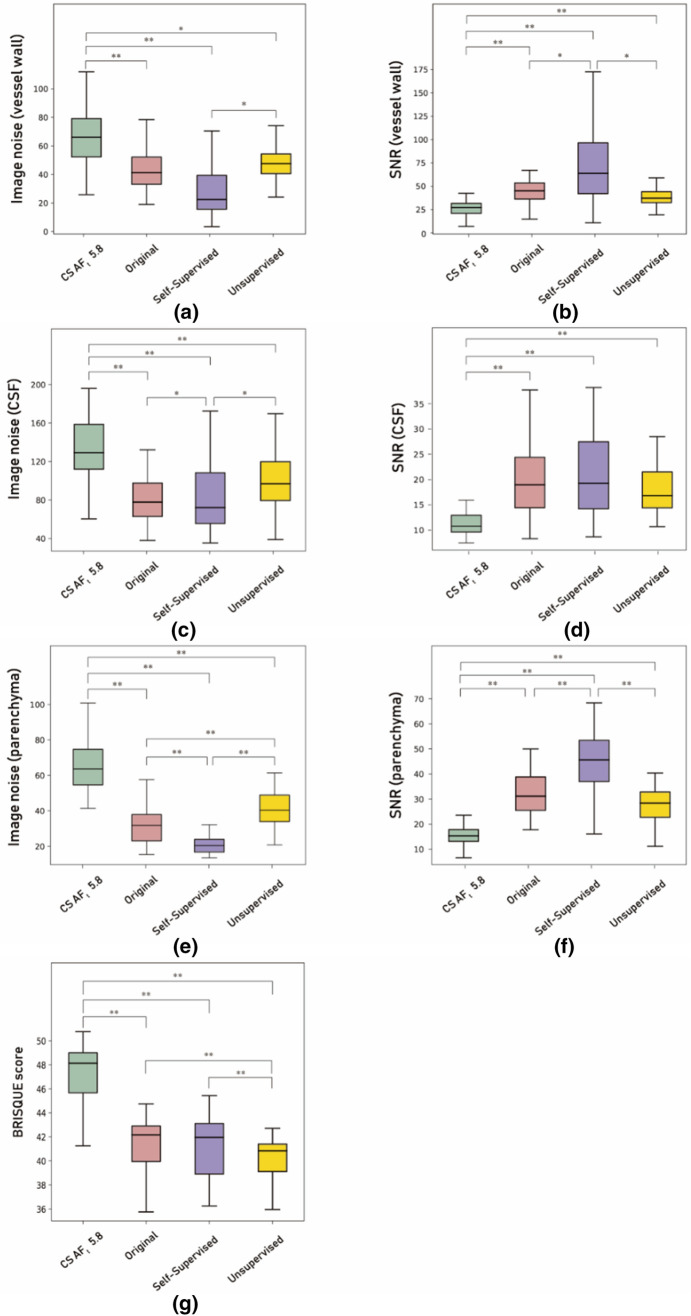
Image noise (**a**, **c**, **e**), signal-to-noise ratio (SNR) (**b**, **d**, **f**), and BRISQUE (**g**) score results of input CS AF_t_ 5.8 images (green), target original images (pink), self-supervised learning output images (purple), and unsupervised learning output images (yellow), at the blood vessel wall (**a**, **b**), CSF (**c**, **d**), and parenchyma (**e**, **f**). Paired *t* test results with **P < 0.01 and *P < 0.05.

The image noise and SNR measurements are depicted in Fig. 10a–f. In both self-supervised and unsupervised learning, the image noise was decreased, and SNR was substantially increased compared with the input CS AF_t_ 5.8 images (*P* < 0.05).

When we compared the original images and the networks’ output images, the image noise of the self-supervised learning output images at the CSF and brain parenchyma was even lower than that of the original output images (*P* < 0.05). The SNR was also improved over that of the original images, except for in the CSF (*P* < 0.05). That is, the self-supervised learning output images had better image quality than the original images in terms of noise and the SNR.

None of the unsupervised learning output images were better than the original images (*P* > 0.05). In brain parenchyma, unsupervised learning output images were significantly inferior to the original images in terms of image noise and SNR (*P* < 0.01).

Furthermore, when we compared the SNR and noise at the vessel wall and brain parenchyma between the self-supervised and unsupervised learning output images, the SNR and noise were markedly better in the self-supervised learning output images (*P* < 0.05).

#### BRISQUE score results

The BRISQUE scores of the input (CS AF_t_ 5.8), target (original), and output images of self-supervised and unsupervised learning are depicted in Fig. 10g. As described in Fig. 10, the BRISQUE scores of both self-supervised and unsupervised learning output images were lower than those of the input images (*P* < 0.01). That is, the output images were seen to be more natural owing to reduced noise^52^ compared with noisy input images. The difference between the original and unsupervised learning outputs was statistically meaningful (*P* < 0.01) and equal to approximately 1 point, whereas the difference between the input and target images was approximately 6 points.

#### Radiomic feature analysis

**Figure 11:**
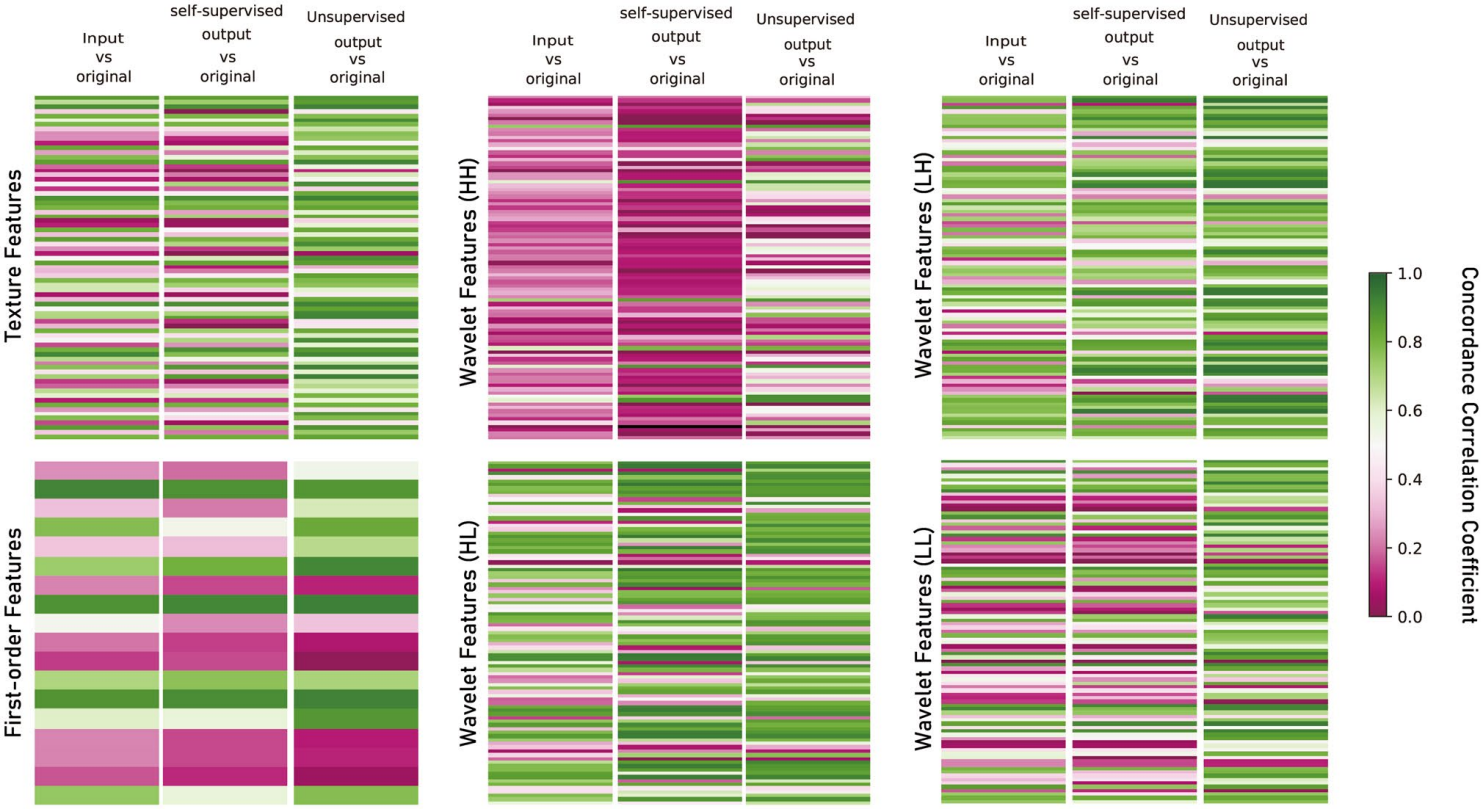
Concordance correlation coefficient (CCC) heat map of radiomics features. While CCCs between self-supervised learning output and original images remain relatively unchanged compared with those between input CS AF_t_ 5.8 and output images, those between unsupervised output and original images improved notably.

The CCCs between the input CS AF_t_ 5.8 and original images, self-supervised output and original images, and unsupervised output and original images are presented in Table 3 and Fig. 11. After image quality enhancement, the CCCs between the output and original images were significantly improved for texture features and wavelet features in the unsupervised learning method (*P* < 0.01), whereas the changes were unknowable in the self-supervised learning method (*P* > 0.05).

**Table 3:** Mean concordance correlation coefficient (CCCs) ± standard deviation between input CS AF_t_ 5.8 and original images, unsupervised output and original images, and self-supervised output and original images. Paired *t* test results were indicated with **P < 0.01.

	Input CS AF_t_ 5.8 vs original images	Supervised output vs original images	Unsupervised output vs original images
Texture features	0.50 ± 0.28	0.47 ± 0.30	0.71 ± 0.20**
First-order features	0.49 ± 0.28	0.43 ± 0.30	0.53 ± 0.35
Wavelet features	0.47 ± 0.28	0.47 ± 0.33	0.62 ± 0.28**

**Figure 12:**
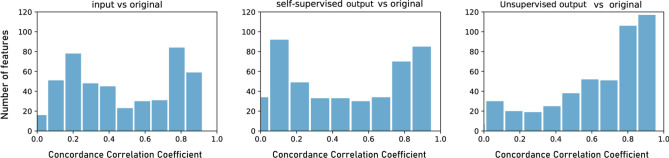
Distribution of concordance correlation coefficient (CCC) between input and original images, unsupervised learning output and original images, and self-supervised learning output and original images.

Among 465 radiomics features, 86 features (18.5%) were reproducible in the input CS AF_t_ 5.8 images and original images (based on a CCC > 0.8^59^, including 17 out of 75 texture features, 3 out of 18 first-order features, and 66 out of 380 wavelet features (Fig. 12). For unsupervised learning output images, a total of 178 features (38.3%) were reproducible including 33 of 75 texture features, 6 out of 18 first-order features, and 139 out of 380 wavelet features. In the case of self-supervised learning output images, a total of 115 features (24.7%) were reproducible including 16 out of 75 texture features, 4 out of 18 first-order features, and 95 out of 380 wavelet features.

#### Visual scoring on high-acceleration CS MR images by neuroradiologist

In terms of image quality, the mean visual scores were 1.00 for the input images, 1.92 for the output images (self-supervised images, 2.00; unsupervised images, 1.83), and 2.50 for the target images. The target images were significantly superior to the input and output images in terms of image quality, and the output images were also significantly superior to the input images in terms of image quality (*P* < 0.05). However, there were no significant differences in image quality between the self-supervised and unsupervised images (*P* > 0.05).

In the vessel delineation, the mean scores were 1.67 for the input images, 2.67 for the output images (self-supervised images, 2.67; unsupervised images, 2.67), and 3.00 for the target images. No significant differences were determined between the output (self-supervised and unsupervised) and target images (*P* > 0.05). The output (self-supervised and unsupervised) and target images were significantly superior to the input images (*P* < 0.05).

## Discussion

In this paper, we presented two DL-based denoising methods for CS HRPD MR images with and without pixel-wise alignments. We demonstrated that both the methods achieved considerable performance in terms of image noise, SNR, BRISQUE scores, radiomic features, and visual scoring, as compared to the input CS AF_t_ 5.8 images, which are not usable in routine clinical practice owing to low image quality^60^. The proposed methods may allow acquisition of more MR images per unit time and may also improve patient comfort. Furthermore, the reduced scan time will result in clearer MR images by avoiding motion artefacts that are commonly introduced by long image acquisition durations.

**Figure 13:**
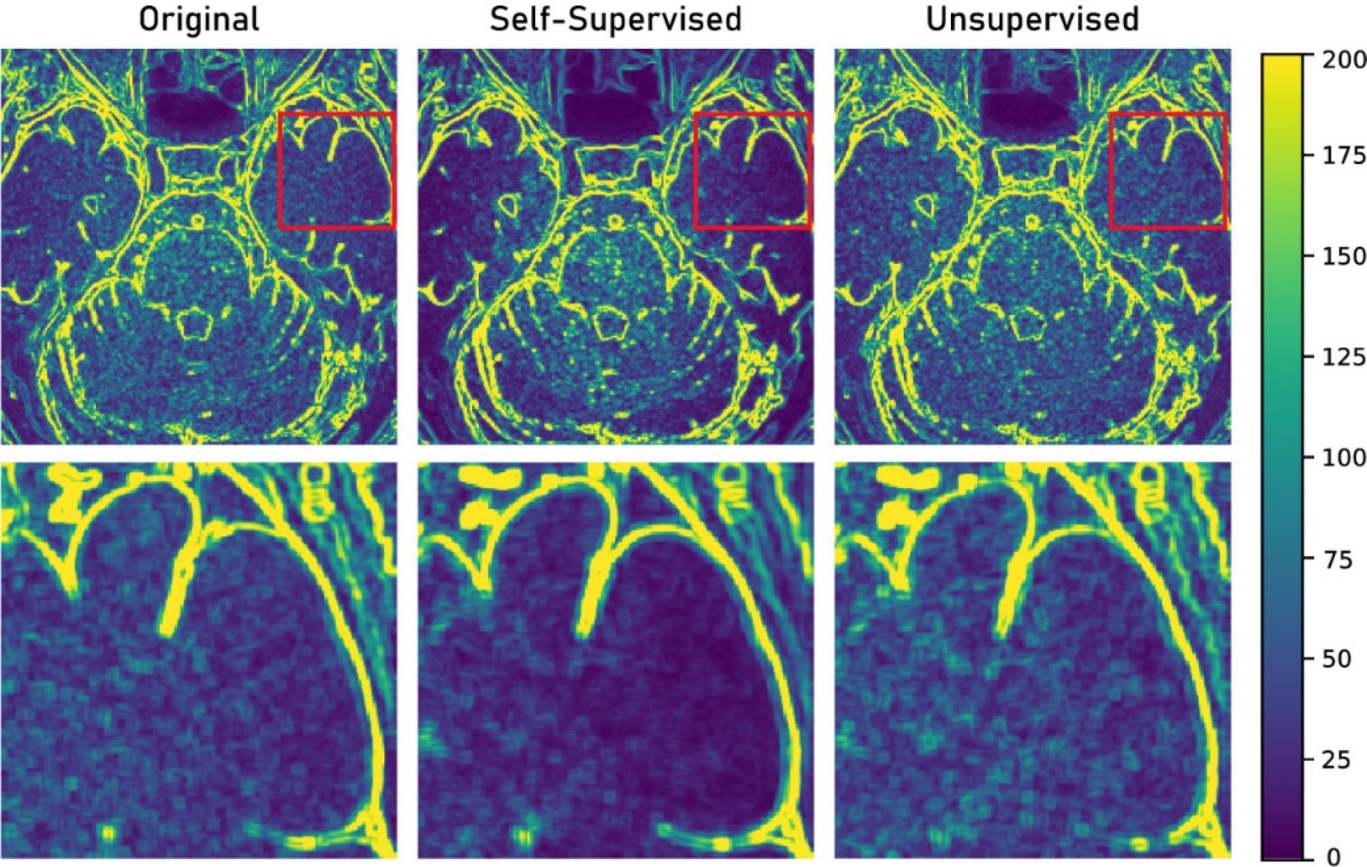
Noise map of original, self-supervised, and unsupervised learning output images. Area in red box in upper row was enlarged in bottom row.

While both methods were promising in terms of image denoising without degradation of image texture, we found that the self-supervised learning outputs were superior in terms of SNR and image noise. In particular, the noise level of brain parenchyma output images obtained with self-supervised learning was even better than those of the target (original) images. This tendency was not observed for the output images of unsupervised learning. Figure 13 displays an example of the noise map, where noise was measured as the standard deviation in every 5 × 5 pixels. The noise pattern of the target image and unsupervised learning output images were similar, as generative adversarial networks learn the data distribution of the target images^61^.

In contrast, the noise within the brain parenchyma was significantly decreased in the output image of self-supervised learning. Two major factors are thought to affect the observed over-denoising in the output images of self-supervised learning: First, the noise distribution of the original MR images was not uniform^62,63^. As the self-supervised network was trained to learn the denoising of uniformly distributed noises, over-denoising could be observed in some regions. Second, the original MR images were not essentially noise-free images. In real clinical practice, noise-free fully sampled MR images is hard to be obtained, as MR data is known to be affected by multiple sources of noise, including thermal noise and electronic noise^22,41^. Therefore, the self-supervised network was indeed trained with noisy–noisy training pairs, rather than noisy–noise-free training pairs. The regression problem we wanted to solve (Eq. (2)) can be expressed as follows:

8 $$ argmin_{\theta } \mathop \sum \limits_{i}^{{}} L\left( {f_{\vartheta } \left( {\widehat{{x_{i} }}} \right),\widehat{{y_{i} }}} \right) $$

where $\widehat{{x_{i} }}$ and $\widehat{{y_{i} }}$ are the noisy input and noisy target, respectively. Although a true clean target $y_{i}$ was not observed, it is still possible to map the corrupted $\widehat{{x_{i} }}{ }$ to the true clean target $y_{i}$ by solving the minimisation task (Eq. (8)) under the condition ${\text{E}}\{ \widehat{{y_{i} }}{ }|\widehat{{x_{i} }}{ }\} { } = { }y_{i}$, which can be met when random noise was introduced to $y_{i}$^32^. As our original images contained random noise, it is not surprising that the self-supervised network outputs were indeed even more denoised than the target original images.

While the self-supervised learning method demonstrates a better result with respect to SNR and noise, the unsupervised learning method improved the reproducibility of radiomics features, whereas the self-supervised learning methods demonstrated no significant advantages in terms of radiomic feature reproducibility. For radiomics researches using multiple medical images with different image qualities, the unsupervised learning method would be advantageous in terms of standardisation and generalisation over the self-supervised learning method. In addition, generating artificial training pairs is not straightforward because of the complex nature of medical images. The unsupervised learning approach could ease this problem because it does not require noise modeling of complex medical images. Accordingly, appropriate DL algorithms should be chosen, depending on the purpose of image translation and the nature of the dataset.

The present study had several limitations. First, the dataset was limited to the CS HRPD MR images of the vessel walls. Further studies should include various image modalities and purposes. Second, our dataset only included the MR images of healthy volunteers. A future study should investigate whether the two different approaches are affected by the clinical diagnosis. Third, this study was limited owing to the small number of subjects as well as the use of a single-vendor MR scanner. In addition, the input CS AF_t_ 5.8 images do not contain residual aliasing artifacts. For further studies, a larger number of subjects, including different types of patients, and various MR scanner types should be included.

## Conclusion

In this paper, we presented and compared two DL-based denoising networks that are applicable to datasets with or without pixel-wise alignments. Both approaches demonstrated promising results when assessed quantitatively and qualitatively. While the self-supervised learning approach was superior to the unsupervised learning approach in terms of SNR and noise level, the unsupervised learning method demonstrated better radiomics feature reproducibility. This study forms the basis for other medical image translation tasks in which pixel-wise alignment is not available.

## Data Availability

Data sharing is not applicable to this article due to medical data privacy protect act.

## Abbreviations

CNN: Convolutional neural network
CS: Compressed SENSE
CSF: Cerebrospinal fluid
CT: Computed tomography
DL: Deep learning
GAN: Generative adversarial network
HRPD: High-resolution proton density-weighted
MR: Magnetic resonance
MRI: Magnetic resonance imaging
MSE: Mean squared error
PD: Proton density-weighted imaging
SENSE: Sensitivity encoding with receiver array
SNR: Signal-to-noise ratio
